# Comparison of the preoperative diagnostic accuracy of BIPSS versus MRI for Cushing disease: a single-centre experience

**DOI:** 10.1186/s12902-023-01277-7

**Published:** 2023-02-16

**Authors:** Jian-Nan Mao, Hui-Ying Yan, Jie-Yu Chen, Chao-Long Yan, Ping Li, Wei Jin, Chun-Hua Hang

**Affiliations:** 1 Department of Neurosurgery, Nanjing Drum Tower Hospital, Clinical College of Nanjing Medical University, Nanjing, People’s Republic of China; 2 Department of Neurosurgery, Nanjing Drum Tower Hospital, Nanjing Medical University, Nanjing, People’s Republic of China; 3 Department of Neuropathology, Nanjing Drum Tower Hospital, Nanjing Medical University, Nanjing, People’s Republic of China; 4 Department of Endocrinology, Nanjing Drum Tower Hospital, Nanjing Medical University, Nanjing, People’s Republic of China

**Keywords:** Cushing syndrome, Cushing disease, Bilateral inferior petrosal sinus sampling, Magnetic resonance imaging, Endoscopic endonasal transsphenoidal surgery

## Abstract

**Background:**

Cushing disease (CD) arises due to a pituitary corticotroph adenoma, which is the most common cause of Cushing syndrome (CS). Bilateral inferior petrosal sinus sampling (BIPSS) is a safe method for differentiating CD from ectopic adrenocorticotropic hormone (ACTH)-dependent CS. Enhanced high-resolution magnetic resonance imaging (MRI) can localize tiny pituitary lesions. The aim of this study was to compare the preoperative diagnostic accuracy of BIPSS versus MRI for CD in CS patients. We performed a retrospective study of patients who underwent BIPSS and MRI between 2017 and 2021. Low- and high-dose dexamethasone suppression tests were performed. Blood samples were collected simultaneously from the right and left catheter and femoral vein before and after desmopressin stimulation. MRI images were obtained, and endoscopic endonasal transsphenoidal surgery (EETS) was performed in confirmed CD patients. Dominant sides of ACTH secretion during BIPSS and MRI were compared with surgical findings.

**Results:**

Twenty-nine patients underwent BIPSS and MRI. CD was diagnosed in 28 patients, 27 of whom received EETS. Localizations of microadenomas by MRI and BIPSS agreed with the EETS findings in 96% and 93% of the cases, respectively. BIPSS and EETS were successfully performed on all patients.

**Conclusion:**

BIPSS was the most accurate method (gold standard) for establishing a preoperative diagnosis of pituitary-dependent CD and was more sensitive than MRI in diagnosing microadenoma. High-resolution MRI with enhancement had an advantage over BIPSS in microadenoma lateralization diagnostics. The combined use of MRI and BIPSS could improve the preoperative diagnosis accuracy in ACTH-dependent CS patients.

## Background

Adrenocorticotropic hormone (ACTH)-dependent Cushing syndrome (CS) commonly occurs due to excessive secretion of ACTH by a pituitary corticotroph adenoma (approximately 70%) or ectopic ACTH-secreting tumour (approximately 30%). Pituitary corticotroph adenomas that cause Cushing disease (CD) are usually too small to visualize. Precise identification and localization of the pituitary tumour responsible for the secretion of ACTH is essential for the surgical treatment of CD. The most reliable procedure for distinguishing CD from ectopic ACTH secretion is bilateral inferior petrosal sinus sampling (BIPSS). However, precise prediction of the lateralization of the pituitary corticotroph microadenoma by BIPSS is sometimes insufficiently reliable. Magnetic resonance imaging (MRI) is widely considered the most accurate imaging modality for ACTH-secreting pituitary adenomas, although a substantial number of patients with CD and biochemical hypercortisolemia still show no visible adenoma on MRI scans. The purpose of this study was to evaluate the diagnostic accuracy of BIPSS and MRI for CD.

## Methods

### Study design and participants

We performed a retrospective study and included patients with ACTH-dependent CS at Nanjing Drum Tower Hospital (NDTH), the Affiliated Hospital of Nanjing University Medical School, in Nanjing, China, between 2017 and 2021. The hospital’s ethics committee approved the study protocol.

### Inclusion and exclusion criteria

The inclusion criteria of this retrospective study were as follows: 1) patients between 20 and 75 years of age with 2) confirmed CS and an unclear ACTH source, 3) CS caused by an ACTH-secreting tumour or EETS confirmed by postoperative pathology or clinical information, biochemical testing, and surgery; and 4) received MRI and BIPSS before surgical resection. The exclusion criteria were as follows: 1) patients with prior pituitary/intracranial surgery; 2) those with incomplete medical records or records that could not be used to explain the discrepancy, and patients with 3) recurrent CD, 4) silent ACTH adenoma, or 5) a diagnosis of ACTH adenoma on clinical presentation with pituitary apoplexy.

### Study protocol

**Fig. 1:**
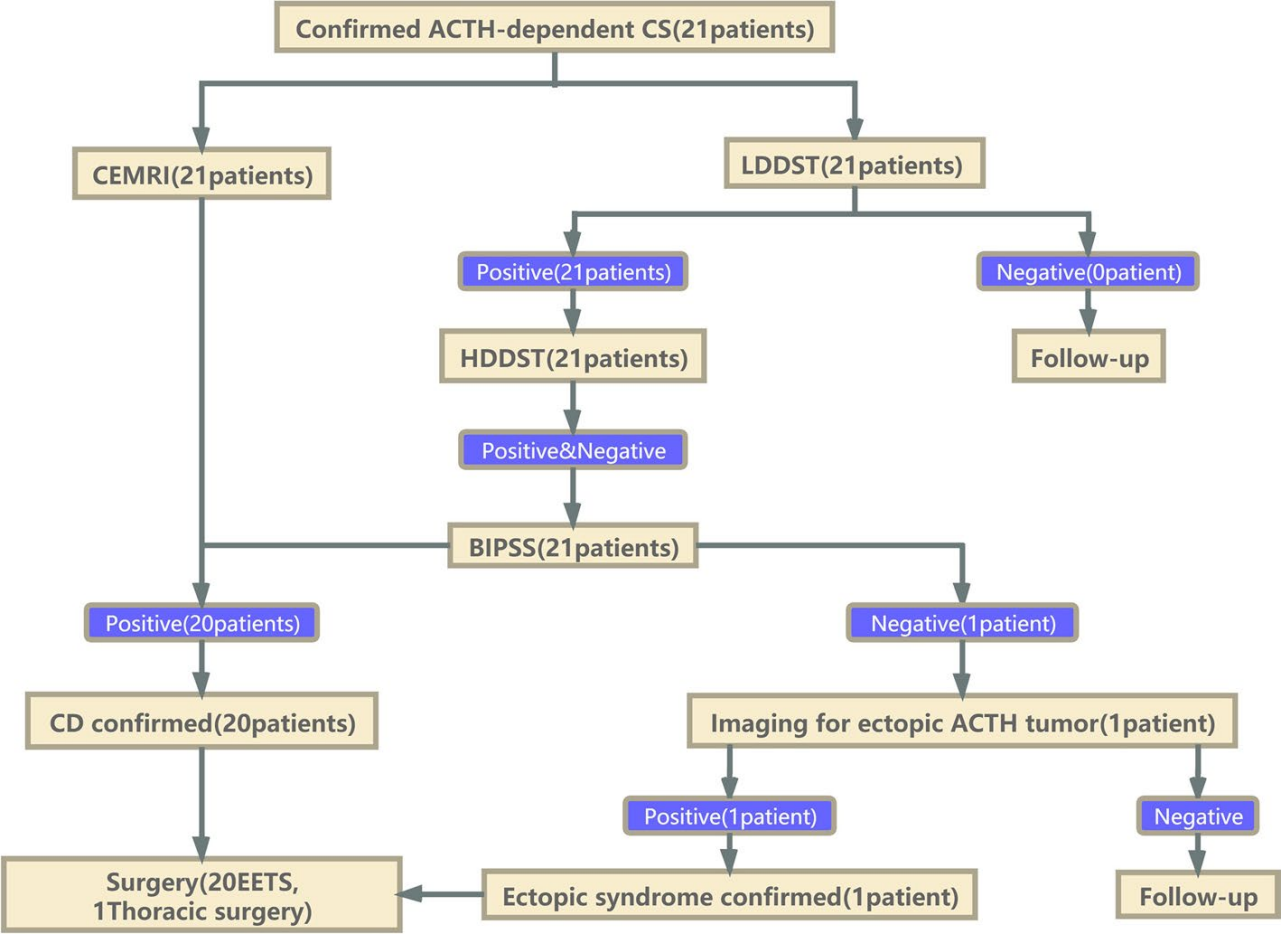
Flowchart for the differential diagnosis of confirmed ACTH-dependent Cushing syndrome and the specific scheme of administration

Clinical information was collected, including age, sex, presenting symptoms, preoperative serum and urinary free cortisol (UFC) levels, ACTH levels, imaging characteristics, technical details of diagnostic procedures, surgical and pathological findings, postoperative hormone levels, subsequent clinical outcomes, and follow-up data. An extensive clinical evaluation was performed on all patients. The diagnosis of CD was made based on standard hormonal criteria and clinical features. All confirmed ACTH-dependent CS patients underwent a contrast-enhanced magnetic resonance imaging (CEMRI) examination and dexamethasone suppression test (DST). Positivity was defined as the high-dose dexamethasone suppression test (HDDST) that could not be inhibited, while negativity was defined when low-dose dexamethasone suppression test (LDDST) could be inhibited. Depending on the positive or negative results, some patients continued follow-up, and the others underwent BIPSS. Those with positive BIPSS results received endoscopic endonasal transsphenoidal surgery (EETS), while those with negative results were further examined (Fig. 1).

### Biochemical tests

When patients demonstrated high levels of cortisol and plasma ACTH (recorded at 12 AM, 8 AM, and 4 PM), routine noninvasive biochemical tests, including low-dose DST (LDDST) (0.5 mg dexamethasone orally every 6 h for 2 days) and high-dose DST (HDDST) (2 mg dexamethasone orally every 6 h for 2 days), were conducted to confirm CS and identify the source of ACTH secretion.

### Imaging

All the patients were examined with pituitary dynamic CEMRI regardless of their LDDST results because some other types of pituitary adenoma can be found using this technique. Patients with ectopic CS underwent additional chest and abdomen contrast-enhanced computerized tomography (CECT). Images were obtained using the same protocol in all patients. We used a 1.5 Tesla or 3.0 Tesla scanner. The coronal and sagittal T1-weighted spin‒echo (SE) sequences before and after intravenous administration of gadolinium-DTPA (0.1 mmol/kg body weight) and T2-weighted sequence on the coronal plane were obtained to achieve the best diagnostic accuracy. Two experienced radiologists, aware of the associated clinical and biochemical information but blinded to the surgical and histopathologic results, independently interpreted the images, which were recorded and classified as follows: positive (a definite small hypointense region in the gland on the enhanced T1-weighted spin‒echo); questionable (indirect signs, such as expansion emerging from the gland, slant pituitary stalk, or a dissymmetric sellar floor, but no clear evidence of a pituitary tumour); or negative (no visible tumour).

### BIPSS protocol

**Fig. 2:**
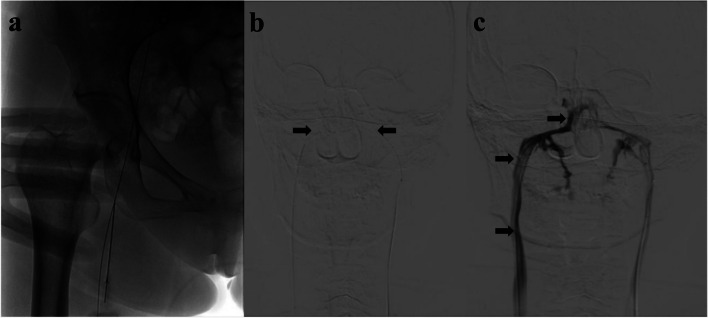
Venography of inferior petrosal sinuses. **a** Puncture of the right femoral vein using the Seldinger technique. **b** Well-positioned catheters within the inferior petrosal sinuses (black arrows). **c** Right inferior petrosal sinus venography during contrast medium injection. The black arrows indicate the inferior petrosal sinus draining directly into the right internal jugular vein

Patients with positive LDDST results received BIPSS. Two 5-French hydrophilic-coated vertebral catheters were used to reach the bilateral petrosal sinuses. The catheters were first introduced into the left and right femoral veins using the Seldinger technique under local anaesthesia (Fig. 2A). Once the catheters were placed in the petrosal sinuses, contrast medium was injected to confirm their position (Fig. 2B, C). The ideal location of the catheters was the tip at the junction of the vertical and horizontal segments of the inferior petrosal sinus. All patients were successfully catheterized via the bilateral inferior petrosal sinuses. Blood samples (8 mL) were collected simultaneously from the right and left catheters and the femoral vein at 0 min (basal level), 5 and 10 min (peak levels) after the administration of 10 µg desmopressin. A ratio of central to peripheral prolactin gradient of 2.0 or greater was also used to verify the correct positioning of the catheter. The ratio of ACTH level in BIPSS to the level of femoral vein samples collected simultaneously was calculated before and after stimulation to diagnose CD. Any side effects during the procedure, such as hypertension, hypotension, bradycardia, tachycardia, headache, internal jugular vein thrombosis, earache, fall in SpO2, nausea, pain in the abdomen, and flushing, were documented.

### EETS protocol

The first and last authors performed EETS on the patients. EETS was performed using a routine two-dimensional endoscopic system. Cerebrospinal fluid leaks were prevented by a mucosal flap with a vascular pedicle, multilayer reconstruction protocols, and postoperative lumbar drainage. The adenoma resection strategy was determined by the preoperative MRI, BIPSS results and by the intraoperative findings. The resection procedures included capsulectomy, en bloc resection, and piecemeal excision. When there was a negative finding on MRI, the bilateral anterior lobe and central portion of the gland were carefully incised and explored [31]. Serum cortisol levels were monitored every hour for three hours after the completion of the surgery (time = 0). Two additional inspections were performed at 24 h and 7 days after the operation. Serum cortisol levels < 5 µg/dL or a 24-hour urinary free cortisol (UFC) level < 20 µg (56 nmol) indicated immediate remission. Any peri-and postoperative adverse events, such as fever, cerebrospinal rhinorrhoea, hypopituitarism, diabetes insipidus, and olfactory impairment, were recorded.

### Immunohistochemistry

**Fig. 3:**
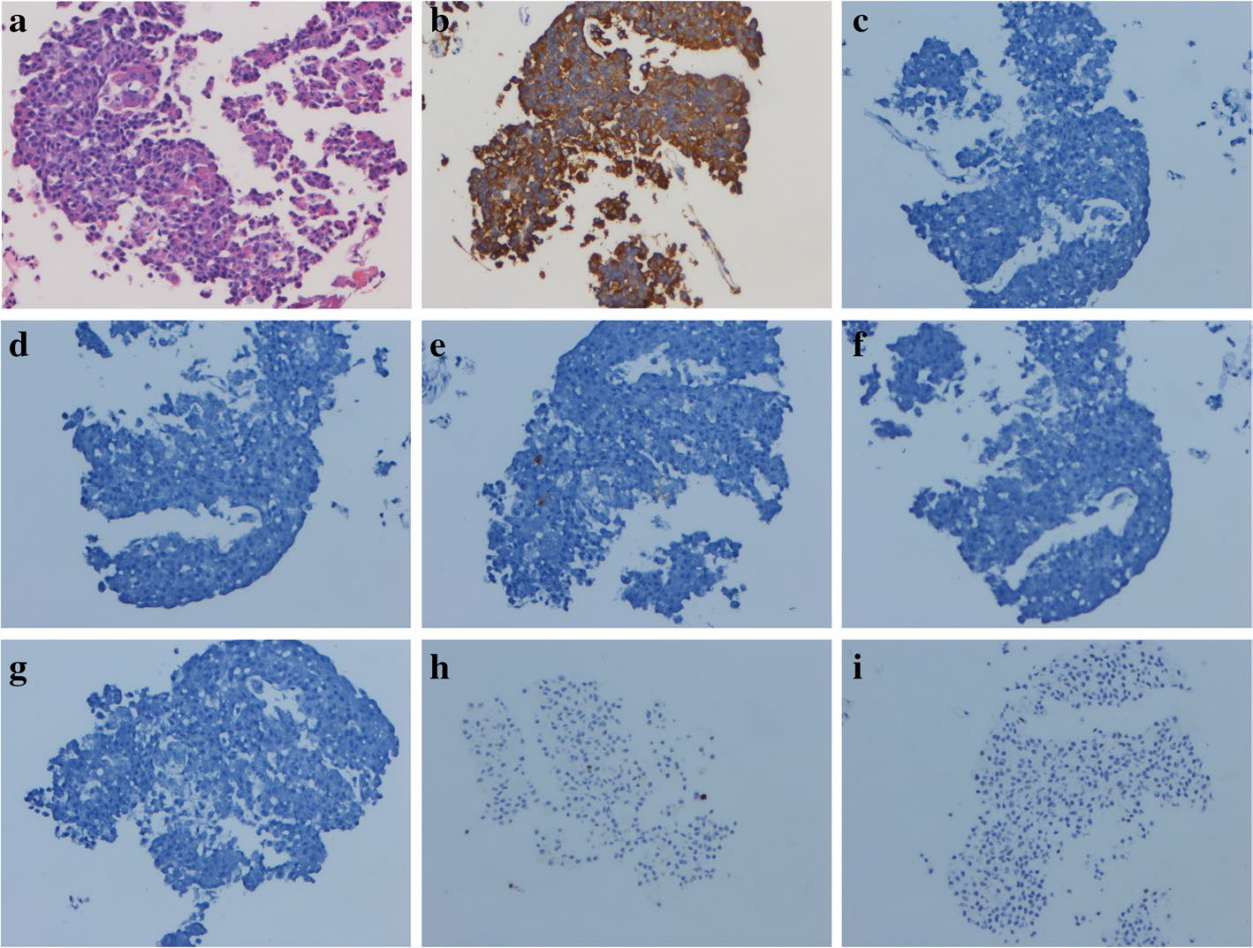
Haematoxylin and eosin staining showing ACTH-secreting pituitary adenoma (**a**). Immunohistochemistry for adrenocorticotropic hormone (**b**), follicle-stimulating hormone (**c**), growth hormone (**d**), prolactin (**e**), luteinizing hormone (**f**), thyroid-stimulating hormone (**g**), Ki67 (**h**), and P53 (**i**)

All specimens were stained with haematoxylin and eosin (HE) to preliminarily confirm the diagnosis of pituitary adenoma. Then, immunohistochemistry for ACTH, growth hormone, thyroid-stimulating hormone, luteinizing hormone, follicle-stimulating hormone, prolactin, Ki67, and P53 was performed on all 19 specimens. Pituitary ACTH-producing adenoma was confirmed if the samples were positive for ACTH on immunohistochemical staining (Fig. 3).

### Statistical analysis

SPSS (version 20.0, IBM Corp, Somers, NY, USA) was used for the statistical analysis. Categorical variables were compared using the pair comparison method. A gold standard for the diagnosis was considered based on the intraoperative localization and lateralization of adenoma. Sensitivity, specificity, predictive values and negative likelihood ratios of BIPSS and CEMRI for the diagnosis of CD were calculated. Concordance between different tests was evaluated.

## Results

This study included 29 consecutively recruited CS patients (23 females, 6 males; mean age, 37.5 ± 19.1 years; age range, 23–73 years). A total of 29 patients underwent BIPSS during the study period. Of these 29 patients, one had a negative BIPSS result, and further abdominal CT showed an ectopic ACTH-producing tumour. CD was confirmed in the remaining 28 patients. The clinical characteristics of these patients and the patient with an ectopic ACTH-producing tumour are shown in Table 1. Twenty-three women and six men (a female-to-male ratio of 3.8:1) with CD were included in the final analysis. The median age of the patients was 37.5 ± 19.1 years. A central/peripheral PRL gradient > 2 was an indicator of successfully sampled central blood. The maximum basal central/peripheral ACTH levels in all 28 patients were > 2. All 28 patients had a central/peripheral ratio > 3 after the desmopressin injection.

**Table 1:** Baseline characteristics and details of BIPSS, pituitary imaging and surgical findings

	BIPSS	MRI	Surgery
% Females	79.3	79.3	81.5
Mean UFC (SD) in nmol/24 h	2284.5 (1223.8)	2284.5 (1223.8)	2287.8 (1251.2)
Mean ACTH (SD) in pmol (8 AM)	23.5 (14.7)	23.5 (14.7)	23.5 (15.1)
Mean ACTH (SD) in pmol (4 PM)	18.2 (11.6)	18.2 (11.6)	17.6 (11.6)
Mean ACTH (SD) in pmol (12 AM)	17.7 (10.7)	17.7 (10.7)	17.7 (11.1)
Mean cortisol (SD) in pmol/24 h after LDDST	20.6 (18.1)	20.6 (18.1)	19.5 (17.8)
Mean cortisol (SD) in pmol/24 h after HDDST	14.8 (14.3)	14.8 (14.3)	13.7 (14.3)
% Right	65.5	34.5	37.0
Mean ratio (SD) of IPS/P before stimulation	12.3 (12.6)	12.3 (12.6)	12.6 (12.9)
Mean ratio (SD) of IPS/P after stimulation (5 min)	10.5 (9.3)	10.5 (9.3)	10.8 (9.3)
Mean ratio (SD) of IPS/P after stimulation (10 min)	7.1 (6.2)	7.1 (6.2)	7.3 (6.2)
Size of pituitary adenoma ≤ 6 mm	15	15	15

*ACTH* Adrenocorticotropic hormone, *LDDST* Low-dose dexamethasone suppression test, *HDDST* High-dose dexamethasone suppression test, *UFC* Urinary free cortisol, *BIPSS* Bilateral inferior petrosal sinus sampling, *MRI* Magnetic resonance imaging, *IPS* Inferior petrosal sinuses, *P* Periphery

**Fig. 4:**
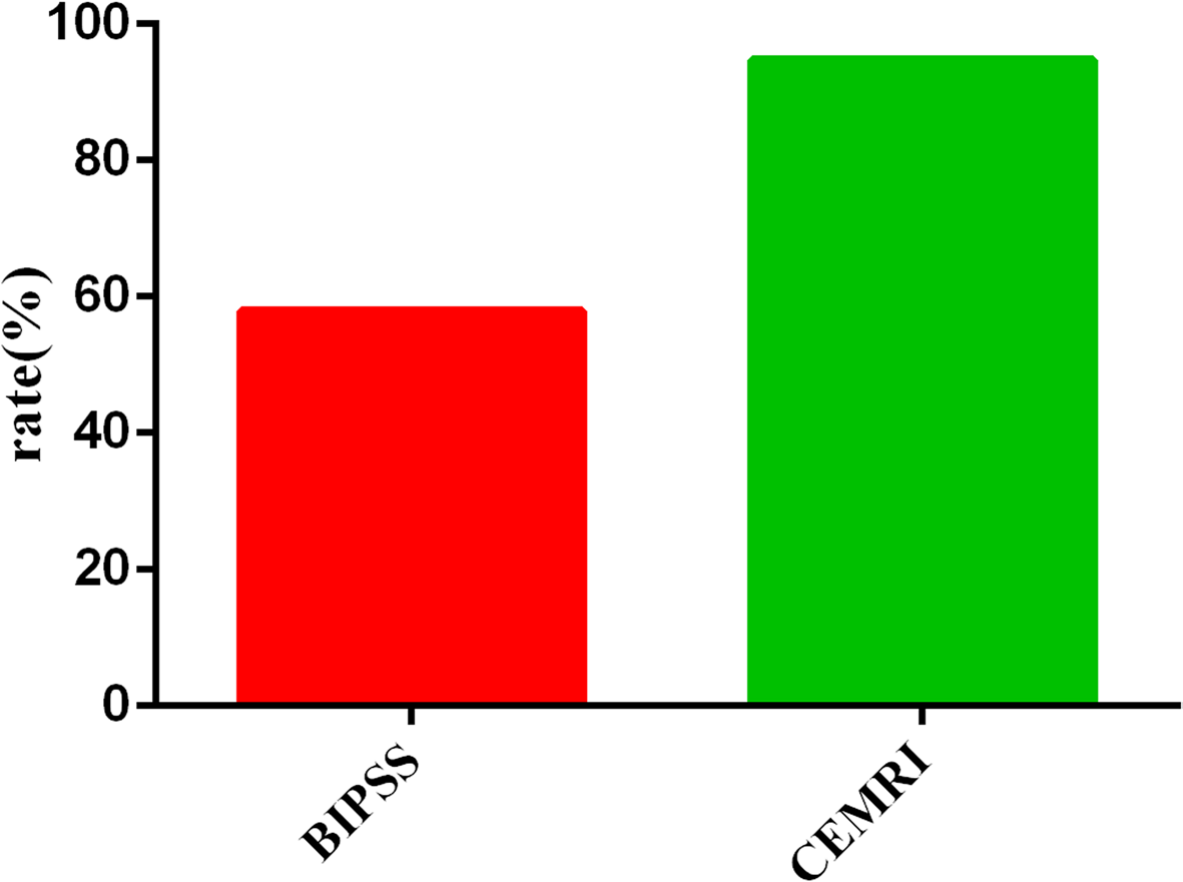
Comparison of diagnostic accuracy between CEMRI and BIPSS

The patient with ectopic CS underwent surgery, which confirmed thymoma. Of the 28 patients with CD, 27 underwent EETS, and 1 received radiation therapy. Table 1 shows details of BIPSS, pituitary imaging, surgical findings, and histological confirmation. The concordance of microadenoma localization by BIPSS, CEMRI, and surgery is shown in Table 2. CEMRI showed microadenoma in 26 patients, while the CEMRI results were normal in the other 3 patients. Nineteen and eight patients showed right and left dominant secretions in BIPSS, respectively. The location of the microadenoma was determined by CEMRI in 26 of the 29 patients (89.7%). Ten lesions were located on the right side of the pituitary gland and 16 on the left; one patient had sheet-like images. The concordances of CEMRI and BIPSS in lateralizing microadenomas were observed in 18 of 28 patients with CD. The concordances in lateralizing microadenoma between BIPSS and EETS were observed in 19 of 27 patients with desmopressin. The concordances of CEMRI with EETS were observed in 26 of 27 patients. The comparison of diagnostic accuracy between CEMRI and BIPSS is shown in Fig. 4. Regarding the diagnostic accuracy of microadenoma localization, the sensitivity, specificity, predictive values, and negative likelihood ratios of BIPSS were 96.4%, 100%, 100%, and 3.6%, respectively. The sensitivity, specificity, predictive values, and negative likelihood ratios of MRI were 92.9%, 100%, 100%, and 7.1%, respectively. Regarding lateralization prediction, the sensitivity of MRI was 96.3%, while the sensitivity of BIPSS was 55.6%.

**Table 2:** Cross-tabulation demonstrating tumor lateralization by BIPSS and CEMRI compared with lateralization by surgery

	Right	Left	Inconclusive
BIPSS	19 (8True, 11False)	8 (7Ture, 1False)	2 (1Ectopic,1Unsure)
CEMRI	10 (10True)	16 (16True)	3 (1Ectopic,2Unsure)
EETS	10	17	2(1TS,1RT)
Concordance (BIPSS/EETS)	15/27 (55.6%)		
Concordance (CEMRI/EETS)	26/27 (96.3%)		

*BIPSS* Bilateral inferior petrosal sinus sampling, *CEMRI* Contrast-enhanced magnetic resonance imaging, *EETS* Endoscopic endonasal transsphenoidal surgery, *TS* Thoracic surgery, *RT* Radiation therapy

True: Same lateralization testified by EETS; False: Different lateralization testified by EETS

The surgical specimens of all 27 patients who underwent EETS were diagnosed by an experienced pathologist, and all 27 were diagnosed with ACTH-producing adenoma. CEMRI and BIPSS are both accurate for diagnosing the pathological type of ACTH-producing adenoma, while BIPSS is ineffective for the pathological type of pituitary adenoma. The specimen was stained with haematoxylin and eosin (HE) to preliminarily confirm the diagnosis of pituitary adenoma (Fig. 3A). Immunohistochemistry for ACTH, growth hormone, thyroid-stimulating hormone, luteinizing hormone, follicle-stimulating hormone, prolactin, Ki67, and P53 was performed to confirm the diagnosis of ACTH-producing adenoma (Fig. 3B) and the samples were negative for markers of the other pathological type (Fig. 3C-I).

**Fig. 5:**
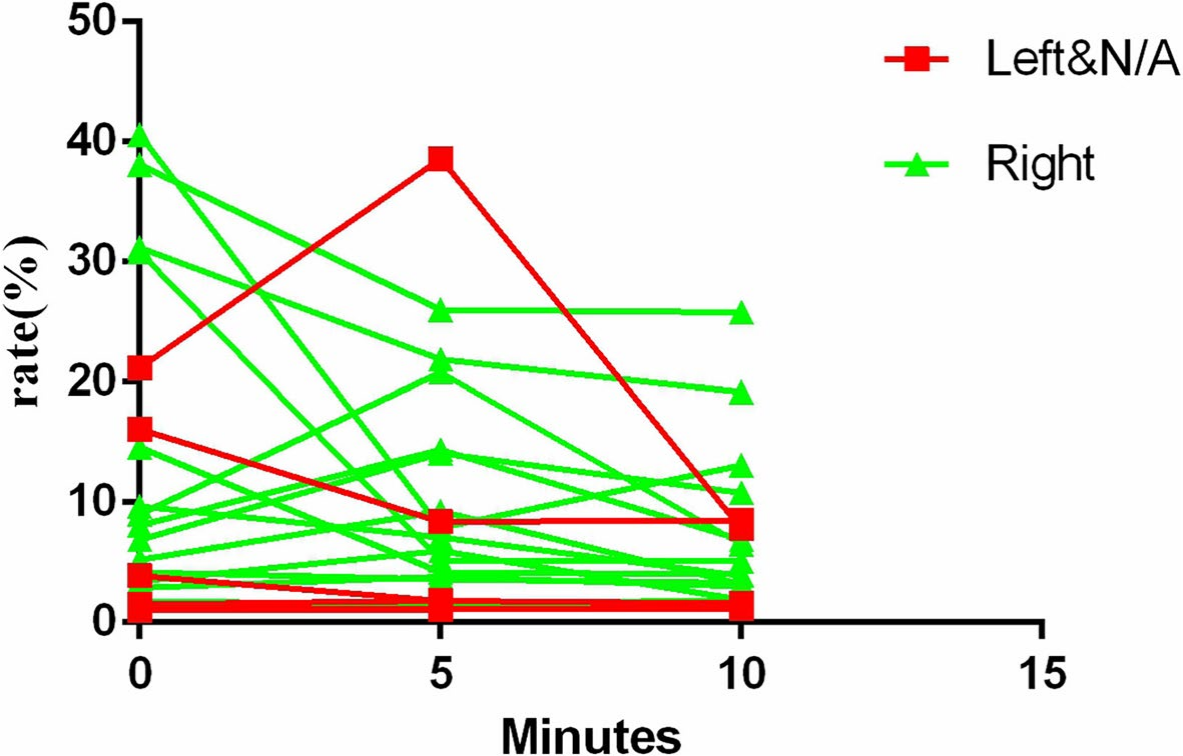
Presentation of the maximal IPS/P ACTH gradient at baseline and after desmopressin administration (5 and 10 min) during the BIPSS procedure

In 15 patients with pituitary adenoma ≤ 6 mm in size, the concordances of CEMRI and BIPSS, BIPSS and EETS, and CEMRI and EETS in lateralizing microadenoma were observed in 8, 8, and 15 patients, respectively. In all 12 patients in which BIPSS was discordant with CEMRI, CEMRI was concordant with intraoperative localization during EETS, suggesting that in the case of discordance between BIPSS and CEMRI, CEMRI could better correlate with the lateralization of adenoma during EETS. The presentations of maximal IPS/P ACTH gradients at baseline and after desmopressin administration (5 and 10 min) during the BIPSS procedure in 29 patients are shown in Fig. 5.

The BIPSS procedure was technically successful (overall catheterization success rate of 100%), with no side effects observed in the perioperative period, such as hypertension, hypotension, bradycardia, tachycardia, headache, internal jugular vein thrombosis, earache, fall in SpO2, nausea, pain in the abdomen, or flushing. All 27 patients successfully underwent EETS, with no fever or cerebrospinal rhinorrhea, while 3 patients (11.1%) had postoperative hypopituitarism and 2 (7.4%) had transient diabetes insipidus during the perioperative period.

## Discussion

BIPSS was first introduced to compare central and peripheral plasma ACTH levels in the early 1980s [26, 28]. Blood samples are obtained from the peripheral vein and simultaneously from both sinuses with central catheters. Although the accuracy of BIPSS in clinical practice does not reach 100%, it remains the best test to differentiate pituitary microadenoma from ectopic ACTH-dependent CS. BIPSS is recommended in the diagnostic workup for CS when the results of clinical, biochemical, and radiologic tests are equivocal or discordant [2]. Although BIPSS is an invasive procedure, the occurrence of adverse events is extremely rare. Since BIPSS does not differentiate pseudo-Cushing or normal states from patients with mild or episodic CD, it must be reserved for patients with clear clinical and biochemical evidence of sustained hypercortisolism due to microadenoma. A central to peripheral ACTH ratio of 3.0 or higher suggests CD [27]. Numerous studies have confirmed the role of BIPSS in the differential diagnosis of CS [3, 4, 20, 29, 34]. At present, BIPSS after desmopressin stimulation is the gold standard for differentiating pituitary from ectopic sources of ACTH. Most patients with ectopic ACTH secretions had a central to peripheral ACTH ratio below 2.0 before and after desmopressin administration.

In our study, 28 patients had a central/peripheral ACTH ratio > 3, and 1 had a central/peripheral ACTH ratio < 3, indicating that the sensitivity of BIPSS was 96.4%. One patient had an ectopic ACTH-producing tumour, which implied a specificity of BIPSS of 100%. Some previous studies have demonstrated a specificity rate of BIPSS between 92.1 and 95% [29, 34]. Oldfield et al. reported in an early study that BIPSS demonstrated 100% sensitivity and specificity in a large patient population [27]. In more recent years, after considering the false-negative and false-positive results, the sensitivity and specificity of BIPSS were determined to be 82–100% and 62.5–100%, respectively [11, 21, 23, 27, 32, 35]. The discrepancy between our data and those in the literature may be due to the limited sample size. We also found the weakness of BIPSS in predicting microadenoma lateralization compared to MRI. Since false-negative results with BIPSS are significantly more common than previously recognized, a negative BIPSS result does not necessarily exclude a pituitary-sourced CS. Desmopressin is a synthetic analogue of human vasopressin that replaces CRH in the BIPSS protocol. Theoretically, overexpression of V2 desmopressin receptors in corticotropin adenoma cells permits desmopressin to stimulate ACTH secretion by binding to pituitary vasopressin receptors [30]. The expression of V2 and V3 receptors has been found in some cases of ectopic CS [1, 9]. However, in our patient with an ectopic ACTH-producing tumour, we did not observe an ascending ACTH level ratio following desmopressin stimulation in either the central or peripheral blood samples. This finding might be attributed to the small quantity of arginine vasopressin (AVP) receptors in some corticotroph adenomas.

Predicting the side of microadenoma in patients with negative imaging is another advantage of BIPSS. The accuracy of BIPSS in lateralizing the lesion was 55.6% in our patient cohort, similar to that in previous reviews, and the accuracy ranged from 54 to 88% [6, 24, 29]. Some studies have reveale much lower accuracy of BIPSS in lateralization of approximately 50–60% [7, 8]. In contrast, other authors have suggested that BIPSS is the best method for localizing microadenomas [17, 22, 25]. The limitation of the previous study [17] was the small group of participants (19 patients).

Numerous factors, including anomalous intercavernous sinus venous connections and catheter position, extension of the epicentre of the tumour to the opposite side, asymmetry or hypoplastic petrosal sinus anatomy, and most importantly, the skill and experience of the interventional team, can influence the accuracy rate of pituitary adenoma lateralization. Anatomical abnormalities of the inferior petrosal sinuses resulting in asymmetrical venous drainage remain the most common cause of false lateralization. Doepp et al. reported that side-to-side analysis resulted in a higher detection rate of IPS signals and higher blood flow velocities on the right side [10]. This result was compatible with the known right-sided dominance of the cerebral venous outflow [12], particularly that of the inferior petrosal sinuses (IPS) [14]. We also found a right-sided dominance of the IPS (a right-to-left ratio of 2.36:1) (Fig. 5). Thus, surgeons must pay attention to the limitations of BIPSS during lateralization.

MRI is commonly used to investigate cranial diseases and remains the optimal technique for noninvasively identifying pituitary microadenomas. Most pituitary adenomas can be found due to improved MRI technology. However, MRI tomographic identification of pituitary ACTH-secreting microadenomas remains problematic. Kaltsas et al. reported that the accuracy of MRI in detecting ACTH-secreting pituitary microadenomas was 60% [18], while Loannis et al. reported an accuracy of 54.3% [19]. Our MRI results showed an accuracy of 96% for the diagnosis of CD. Because of the small size of microadenomas, the enhancement characteristics are similar to those of the normal pituitary gland. Therefore, ACTH-secreting microadenomas are frequently invisible on MRI in patients with CD. The dynamic technique allowed us to achieve high sensitivity and specificity with the injection of intravenous gadolinium in multiple coronal and sagittal sequences. A microadenoma could be found by this technique in 96% of patients with a biochemical diagnosis of CD [13]. In suspected ACTH-dependent CD patients, adenomas could be identified with a 50–60% higher diagnostic sensitivity rate by dynamic versus nondynamic MRI.

Another limitation of MRI identification for CD is false-positive results. Blurring artefacts or pituitary incidentalomas might be accountable for these false-positive results [33]. Approximately 10% of the healthy adult population has pituitary abnormalities on MRI scans that are considered asymptomatic microadenomas [16].

Endoscopic endonasal approach selective adenectomy is considered the ideal treatment for patients with CD, with a high possibility of remission and low complication rates [5, 15]. For patients with clear lateralization on CEMRI, the lesions could be resected completely via an endoscopic endonasal approach. However, in those with negative preoperative MRI results, the EETS performs equally to planned excision on the side determined by BIPSS. If no microadenoma is identified during surgery, some surgeons propose performing a hemi-hypophysectomy on the side where BIPSS detected the gradient or the side with an obvious or suspicious CEMRI finding [31]. Reppucci et al. suggested performing a hemihypophysectomy on the side of the CEMRI abnormality when CEMRI shows a suspicious finding that contradicts the BIPSS findings and no microadenoma is identified during the surgery [31]. All microadenomas were clearly observed in our study, and EETS was performed based on the indication of CEMRI or BIPSS. No hemihypophysectomy was adopted. No severe complications, such as diabetes insipidus, cerebrospinal fluid (CSF) leaks, anterior/posterior pituitary insufficiency, nasal bleeding, local infection, or visual damage, were observed in any of the patients. However, some patients developed olfactory impairments after the operation. Most of the patients recovered after treatment with neurotrophic drugs in the subsequent months.

## Conclusion

BIPSS was the most accurate method (gold standard) for establishing a preoperative diagnosis of pituitary-dependent CD and was more sensitive than MRI for diagnosing microadenoma. High-resolution MRI with enhancement had an advantage over BIPSS in microadenoma lateralization diagnostics. The combined use of a multimodality diagnostic approach, including clinical presentations, biochemical tests, MRI, and BIPSS, could improve the accuracy of preoperative diagnostics.

## Data Availability

The data used in present study are available by corresponding author on request.

## Abbreviations

BIPSS: Bilateral inferior petrosal sinus sampling
CD: Cushing disease
CS: Cushing syndrome
ACTH: Adrenocorticotropic hormone
MRI: Magnetic resonance imaging
EETS: Endoscopic endonasal transsphenoidal surgery
NDTH: Nanjing Drum Tower Hospital
UFC: Contrast-enhanced magnetic resonance imaging
DST: Dexamethasone suppression test
HDDST: High-dose dexamethasone suppression test
LDDST: Ligh-dose dexamethasone suppression test
CECT: Contrast-enhanced computerized tomography
SE: Spin‒echo
HE: Haematoxylin and eosin
AVP: Arginine vasopressin
CSF: Cerebrospinal fluid
